# Laboratory and Simulated Field Bioassays to Evaluate Larvicidal Activity of *Pinus densiflora* Hydrodistillate, Its Constituents and Structurally Related Compounds against *Aedes albopictus*, *Aedes aegypti* and *Culex pipiens pallens* in Relation to Their Inhibitory Effects on Acetylcholinesterase Activity

**DOI:** 10.3390/insects4020217

**Published:** 2013-05-30

**Authors:** Dong Chan Lee, Young-Joon Ahn

**Affiliations:** 1 International Course, Hankuk Academy of Foreign Studies, Yongin 449-854, Korea; E-Mail: stjohndlee@hafs.hs.kr; 2 WCU Biomodulation Major, Department of Agricultural Biotechnology, Seoul National University, Seoul 151–921, Korea

**Keywords:** *Pinus densiflora*, botanical larvicide, disease vector mosquitoes, insecticide resistance, AChE inhibition

## Abstract

The toxicity of *Pinus densiflora* (red pine) hydrodistillate, its 19 constituents and 28 structurally related compounds against early third-instar larvae of *Aedes albopictus (Ae. albopictus)*, *Aedes aegypti* (*Ae. aegypti*) and *Culex pipiens palles* (*Cx. p. pallens*) was examined using direct-contact bioassays. The efficacy of active compounds was further evaluated in semi-field bioassays using field-collected larval *Cx. p. pallens*. Results were compared with those of two synthetic larvicides, temephos and fenthion. In laboratory bioassays, *Pinus densiflora* hydrodistillate was found to have 24 h LC_50_ values of 20.33, 21.01 and 22.36 mg/L against larval *Ae. albopictus*, *Ae. aegypti and Cx. p. pallens* respectively. Among the identified compounds, thymol, *δ*-3-carene and (+)-limonene exhibited the highest toxicity against all three mosquito species. These active compounds were found to be nearly equally effective in field trials as well. *In vitro* bioassays were conducted to examine the acetylcholinesterase (AChE) inhibitory activity of 10 selected compounds. Results showed that there is a noticeable correlation between larvicidal activity and AChE inhibitory activity. In light of global efforts to find alternatives for currently used insecticides against disease vector mosquitoes, *Pinus densiflora* hydrodistillate and its constituents merit further research as potential mosquito larvicides.

## 1. Introduction

The task of controlling mosquito populations is of the utmost importance in the fields of public health because mosquitoes serve as the major vector for many diseases such as malaria, dengue fever, filariasis and yellow fever [1]. In 2004, it was estimated that approximately 3.2 billion people were exposed to the risks of malaria infection and that some 350–500 million people contracted the disease, with at least a million deaths incurred annually [2]. Nearly 925 million people in tropical regions live in the dangers of dengue infection [3]. *Aedes albopictus (Ae. albopictus)* and *Aedes aegypti* (*Ae. aegypti*) are two main species of mosquitoes responsible for the transmission of dengue fever, Chikungunya and yellow fever, and *Culex pipiens palles* (*Cx. p. pallens*) carry Japanese encephalitis, meningitis and West Nile virus.

Mosquito larvae control against these species has been achieved mainly by the use of organophosphorus (OP) insecticides, insect growth regulators and bacterial larvicides [4]. However, indiscriminate use of these larvicides has disrupted natural biological control systems and led to resurgence of mosquitoes [5] and has often resulted in widespread development of resistance [6]. Increasing levels of resistance to commonly used insecticides have invariably led to multiple treatments and excessive doses, posing serious threats to both the environment and human health. In light of such trends around the globe, the urgent need for the development of selective mosquito control alternatives that can help to procure an effective resistance management strategy should be addressed adequately and promptly.

As alternative sources for disease vector control products, plant extracts have drawn a great deal of attention because they are considered to be a potential source of bioactive chemical compounds that are relatively safe with few side effects on the environment and human health [7,8,9]. They often act at various novel target sites [10,11] and thus significantly reduce the possibility of resistance development in mosquitoes.

Recently, it was found out that the hydrodistillate of red pine (*Pinus densiflora*) needles and their active compounds exhibit high toxicity against house dust mites (*Dermatophagoides farinae*) and have the potential of being developed into commercial insecticides against house dust mites [12]. However, no information was yet available regarding the potential insecticidal activity of *Pinus densiflora* against mosquitoes.

In the present study, the potential toxicity of *P. densiflora* hydrodistillate (PD-HD), its 19 constituents [12] and 28 structurally related compounds against early third-instar larvae of *Ae. albopictus*, *Ae. aegypti* and *Cx. p. pallens* was evaluated and then compared with that of two synthetic larvicides, temephos and fenthion. Semi-field bioassays were conducted using field-collected larval *Cx. p. pallens*. In addition, 10 compounds were selected from among all the compounds tested for their larvicidal activity, and their inhibitory effects on acetylcholinesterase (AChE) activity were further examined with the aim of developing a deeper understanding of their modes of action.

## 2. Materials and Methods

### 2.1. Chemicals

A total of 47 commercially available pure organic compounds examined in this study are listed in Table 1, with their sources indicated accordingly. Temephos (97.3% [AI]) and fenthion (98.4% [AI]) were purchased from Riedel (Seelze, Germany) and Supelco (Bellefonte, CA, USA) respectively. Triton X-100 was purchased from Shinyo Pure Chemicals (Osaka, Japan). All the other chemicals used in this study were of a reagent-grade quality and were commercially available.

**Table 1:** 47 commercially available pure organic compounds examined in this study.

Compound	Source	Compound	Source
*α*-humulene *^a,c^*	TCI *^d^*	Caryophyllene oxide *^a^*	S-A
*α*-terpinene *^a^*	S-A *^e^*	1,8-cineole *^c^*	S-A
Carveol *^c^*	S-A	Citral *^c^*^,^*	S-A
Nerol	TCI	*p*-cymene	S-A
(*S*)-(+)-carvone	S-A	Geraniol	S-A
(+)-*β*-citronellol	S-A	Geranyl acetate	WK *^h^*
(−)-*β*-citronellol	S-A	(+)-limonene *^a^*^,*b*^	TCI
(+)-citronellal	FA *^f^*	Linalool *^a^*	S-A
(−)-citronellal	FA	Linanyl acetate *^c^*	WK
(−)-myrtenal *^c^*	S-A	Menthol *^c^*	S-A
(−)-cis-myrtanol	S-A	*β*-myrcene *^a^*	TCI
(−)-myrtenol	SC *^g^*	*α*-phellandrene *^a^*	TCI
Carvacrol	S-A	(1*R*)-(+)-*α*-pinene *^a^*	TCI
(−)-verbenone	FA	(1*S*)-(−)-*α*-pinene *^a^*	S-A
(*S*)-cis-verbenol	S-A	(1*R*)-(+)-*β*-pinene *^a^*	S-A
*o*-cymene	S-A	(1*S*)-(−)-*β*-pinene *^a^*^,*b*^	S-A
*m*-cymene	S-A	*γ*-terpinene *^a^*	S-A
Aromadendrene *^a^*	S-A	*α*-terpineol	S-A
Borneol *^a^*	S-A	(+)-terpinen-4-ol	TCI
Bornyl acetate *^a^*	S-A	(−)-terpinen-4-ol	TCI
Camphene *^a^*	S-A	*α*-terpinolene *^a^*^,*b*^	TCI
Camphor	S-A	Terpinyl acetate	TCI
*δ*-3-carene *^a^*^,*b*,*c*^	S-A	Thymol *^b^*^,*c*^	S-A
*β*-caryophyllene *^a^*^,^*^c^*	TCI		

*^a^* Constituents of *Pinus densiflora* as reported by Lee *et al.* [12]. *^b^* Compounds tested in semi-field bioassays. *^c^* Compounds tested in AChE inhibition assays. *^d^* Purchased from Tokyo Chemical Industry (Tokyo, Japan). *^e^* Purchased from Sigma-Aldrich (St Louis, MO). *^f^* Purchased from Fluka (Buchs, Switzerland). *^g^* Purchased from SAFC Supply Solutions (St. Louis, MO, USA). *^h^* Purchased from Wako (Osaka, Japan). * Neral: Geranial = 1:1.

### 2.2. Hydrodistillate Preparation

Fresh *P. densiflora* needles were collected at Mt. Gwanak (Seoul) in early July, 2012. The needles (500 g) were finely ground by a blender and were subjected to hydrodistillation at 100 °C for 6 h, using Clevenger-type apparatus. The volatile oil was dried over anhydrous sodium sulfate and was stored in a sealed vial at 4 °C until use. The yield of the hydrodistillate was 1.11% based on dried weight of the plant.

### 2.3. Mosquitoes

Stock cultures of *Ae. albopictus*, *Ae. aegypti* and *Cx. p. pallens*, originally obtained from the National Institute of Health, Korea Centers for Disease Control and Prevention (Seoul, Korea) in 1999, were maintained in the laboratory without any exposure to any known insecticide. Adult mosquitoes were maintained on a 10% sugar solution and blood fed on live mice. Larvae were reared in plastic trays (24 by 35 by 5 cm) containing 0.5 g of sterilized diet (40-mesh chick chow powder/yeast, 4:1 by weight). All three species were reared at 26–28 °C, 65–75% RH, and a photoperiod of 16:8 (L:D) h.

**Figure 1:**
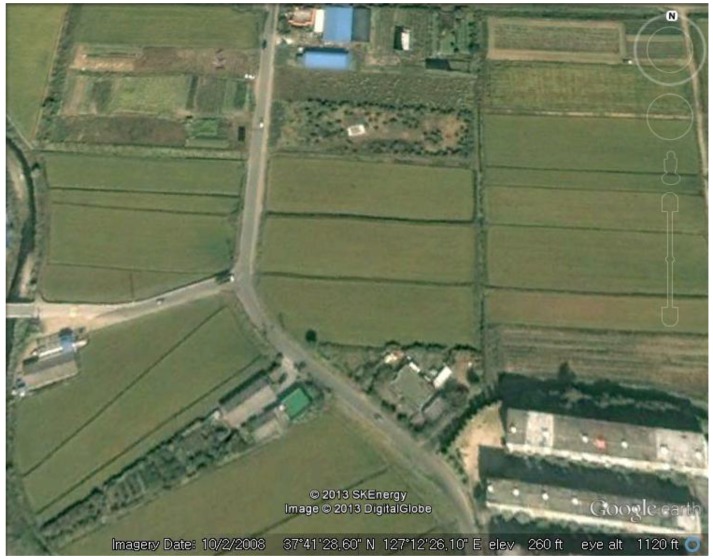
A satellite view of *Cx. p. pallens* larvae collection site & venue for semi-field bioassays.

Larvae of wild mosquitoes were field-collected from irrigated rice fields in Yongin, Korea in early August 2012. A satellite view of the larvae collection site is included in Figure 1 [13]. Through species identification based on polymerase chain reaction (PCR) [14], it was revealed that the collected larvae belong to *Cx. p. pallens.*

### 2.4. Bioassays

Direct-contact mortality bioassays [15] were employed to evaluate the toxicity of all compounds, using early third-instar larvae of each mosquito species. Each compound was dissolved in methanol, and then further diluted in distilled water containing Triton X-100 (20 µL/L). Groups of 20 mosquito larvae of each species were put into separate paper cups (270 mL), each containing different compound solutions (250 mL). The toxicity of each compound was determined by repeating this experimental procedure with different concentrations, ranging from 5 to 200 mg/L. Temephos and fenthion served as standard references and were similarly formulated as well. Controls contained only methanol-Triton X-100 carrier solution in distilled water.

Both treated and control larvae were kept under the same conditions as those used for colony maintenance. Larvae were considered to be dead only if they did not show any signs of movement when prodded with a fine wooden dowel at 24 h post-treatment. Due to certain constraints arising from the number of available larvae, all necessary bioassays could not be conducted simultaneously and thus, treatments were conducted over a period of time, with a separate control treatment included each time. Freshly prepared compound solutions were used for each round of bioassays [16]. All bioassays were repeated three times.

### 2.5. Semi-Field Bioassays

The efficacy of *P. densiflora* hydrodistillate and its five most toxic constituents as potential larvicides was further verified in semi-field bioassays. For each selected concentration, as guided by the results observed in above-described bioassays, of each test compound, three buckets of water from irrigated rice fields were prepared, and a batch of 20 field-collected larvae of *Cx. p. pallens* was released into each bucket. The buckets were treated with test compounds and covered with nylon mesh screen to prevent other mosquitoes and insects from laying eggs. The buckets were then placed back at the initial collection site and were allowed to stand for 24 h, after which the mortality data were recorded. Temephos and fenthion served as standard references.

### 2.6. AChE Inhibition Assays

Among all the compounds tested for their larvicidal activity, a total of 10 compounds were carefully selected in such a way that their demonstrated LC_50_ values represent a wide scope of evenly dispersed values ranging from some of the lowest (*i.e*., strong toxicity) to some of the highest (*i.e.*, low or no toxicity).

For each species, larvae frozen at −20 °C were decapitated and 100 heads were homogenized in 5 mL of ice-cold 0.1 M sodium phosphate buffer (pH 8.0), using a Teflon glass tissue homogenizer. The homogenate was centrifuged at 10,000 × *g* at 4 °C for 20 min. The supernatant was filtered by a 0.22 µm-Millex-GV filter (Millipore, Cork, Ireland) and was used as the AChE preparation. Protein concentrations were determined by the Bradford dye method [17] using BSA as the standard. A microplate AChE assay was carried out following the method of Moores *et al.* [18] adapted from Ellman *et al.* [19]. The reaction mixture consisted of 80 µL of the crude enzyme preparation, 10 µL of 7.5 mM DTNB in phosphate buffer (pH 7.0) and 100 µL of the selected test compounds of various concentrations in 2.5% acetone. The reaction mixture was incubated at 30 °C for 5 min and 10 µL of 6.25 mM ATChI was then added to the mixture. The absorbance was recorded at 414 nm, using a Molecular Devices VersaMax microplate reader (Sunnyvale, CA, USA). All AChE inhibition assays were repeated three times in triplicates.

### 2.7. Data Analysis

Concentration-mortality data were subjected to probit analysis using SAS [20]. The LC_50_ values of each species, and their treatments, were considered to be significantly different from one another only if their 95% confidence limits failed to overlap. Compounds that have LC_50_ > 150 mg/L were considered to be ineffective. The IC_50_ (the concentration required to inhibit the AChE activity by 50%) values for compounds tested in AChE inhibition assays were determined using GraphPad Prism five (GraphPad Software, San Diego, CA, USA). Furthermore, for the compounds tested in AChE inhibition assays, a correlation analysis between their LC_50_ values and IC_50_ values was conducted using SAS [20].

## 3. Results

### 3.1. Larvicidal Activity of Test Compounds

The toxicity of *P. densiflora* hydrodistillate (PD-HD) and two commercially available synthetic larvicides against early third-instar larvae of three mosquito species was evaluated using a direct-contact mortality bioassay. Against *Ae. albopictus*, *Ae. aegypti* and *Cx. p. pallens*, PD-HD exhibited 24 h LC_50_ values of 20.33, 21.01 and 22.36 mg/L respectively (Table 2). PD-HD was demonstrated to have significantly strong, albeit much weaker than either temephos or fenthion, larvicidal activity, against all of the three species, with LC_50_ values lower than 25 mg/L.

**Table 2:** Toxicity of *Pinus densiflora* hydrodistillate (PD-HD), 19 PD-HD constituents, 28 structurally related compounds and two commercially available synthetic larvicides against early third-instar larvae of *Ae. albopictus*, *Ae. aegypti* and *Cx. p. pallens* after 24 h exposure.

Compound	LC_50 (*Ae. albopictus*)_, mg/L (95% CL *^a^*)	LC_50 (*Ae. aegypti*)_, mg/L (95% CL *^a^*)	LC_50 (*Cx p. pallens*)_, mg/L (95% CL *^a^*)
PD-HD	20.33 (16.49–24.72)	21.01 (17.24–25.31)	22.36 (18.33–27.04)
*α*-humulene	>150	>150	>150
*α*-terpinene	21.88 (18.29–26.24)	24.85 (20.96–29.18)	23.87 (19.63–29.20)
Carveol	121.22 (106.48–143.40)	123.97 (111.02–142.51)	113.58 (101.09–129.69)
Nerol	111.59 (96.03–133.34)	120.83 (104.33–147.41)	111.48 (97.78–129.50)
(*S*)-(+)-carvone	43.83 (41.78–45.91)	42.14 (39.62–44.62)	46.19 (43.54–49.12)
(+)-*β*-citronellol	90.89 (83.26–99.00)	93.46 (85.80–101.82)	87.81 (80.62–95.50)
(−)-*β*-citronellol	71.99 (64.67–80.37)	75.31 (68.31–83.22)	66.26 (59.63–73.33)
(+)-citronellal	46.39 (41.08–52.95)	48.23 (42.42–55.64)	49.47 (43.52–57.04)
(−)-citronellal	41.06 (38.44–43.59)	37.46 (34.19–40.50)	41.90 (39.07–44.69)
(−)-myrtenal	137.86 (119.15–173.91)	>150	139.61 (122.92–169.77)
(−)-cis-myrtanol	>150	>150	>150
(−)-myrtenol	>150	>150	>150
Carvacrol	95.05 (77.66–120.51)	101.06 (84.95–124.54)	91.93 (75.83–114.29)
(−)-verbenone	89.39 (82.78–96.07)	96.78 (89.76–104.20)	89.07 (82.69–95.50)
(*S*)-cis-verbenol	136.86 (121.92–161.87)	>150	140.41 (124.89–167.57)
*o*-cymene	48.57 (42.14–55.77)	39.60 (33.25–46.71)	47.78 (40.96–55.39)
*m*-cymene	62.05 (54.45–70.02)	69.70 (61.83–78.37)	59.35 (52.20–66.67)
Aromadendrene	129.21 (118.02–145.97)	>150	147.91 (127.93–188.26)
Borneol	>150	>150	>150
Bornyl acetate	>150	>150	>150
Camphene	79.09 (68.89–90.41)	72.92 (65.75–80.81)	80.06 (72.21–88.87)
Camphor	>150	>150	>150
*δ*-3-carene	9.33 (8.27–10.38)	12.10 (10.36–13.81)	10.11 (8.72–11.74)
*β*-caryophyllene	39.52 (36.51–42.85)	38.58 (36.17–41.09)	47.79 (41.47–54.25)
Caryophyllene oxide	107.62 (99.82–116.81)	113.00 (104.54–123.59)	126.28 (110.92–158.13)
1,8-cineole	73.50 (69.80–77.70)	73.30 (68.56–77.97)	72.88 (68.99–76.68)
Citral	41.63 (38.71–44.48)	49.19 (45.19–54.06)	47.80 (43.91–51.84)
*p*-cymene	32.83 (28.23–38.63)	33.46 (29.27–37.78)	29.66 (25.22–34.29)
Geraniol	68.17 (64.13–72.27)	73.44 (68.48–78.23)	56.96 (51.05–63.85)
Geranyl acetate	33.01 (30.60–35.49)	21.39 (17.37–25.63)	30.33 (27.17–33.83)
(+)-limonene	10.77 (9.80–11.87)	15.31 (12.69–18.51)	10.76 (9.27–12.54)
Linalool	129.04 (117.54–146.31)	113.47 (101.40–128.73)	144.16 (123.66–186.95)
Linanyl acetate	98.12 (91.30–105.44)	97.76 (90.78–106.32)	96.96 (89.59–104.00)
Menthol	69.61 (64.26–74.80)	81.20 (75.05–87.04)	67.26 (59.30–74.32)
Myrcene	35.98 (30.30–42.14)	39.51 (32.70–46.68)	41.31 (37.50–45.37)
*α*-phellandrene	16.86 (14.43–19.69)	19.35 (16.31–22.66)	14.04 (12.09–16.50)
(1*R*)-(+)-*α*-pinene	55.65 (52.49–59.18)	51.28 (46.32–56.56)	60.84 (58.55–63.28)
(1*S*)-(−)-*α*-pinene	28.61 (26.09–31.65)	39.98 (34.62–46.52)	31.98 (28.39–35.68)
(1*R*)-(+)-*β*-pinene	23.44 (19.88–27.27)	24.06 (20.61–27.80)	24.02 (20.11–28.36)
(1*S*)-(−)-*β*-pinene	15.35 (12.76–18.15)	17.33 (14.35–20.50)	16.17 (13.26–19.75)
*γ*-terpinene	16.25 (12.49–20.32)	20.48 (16.63–24.64)	17.75 (13.89–22.21)
*α*-terpineol	131.79 (120.10–149.75)	>150	106.32 (97.22–115.84)
(+)-terpinen-4-ol	98.28 (91.85–105.07)	78.73 (71.24–85.73)	86.25 (78.22–95.55)
(−)-terpinen-4-ol	92.16 (84.28–101.24)	72.36 (66.33–78.22)	85.66 (75.71–97.28)
*α*-terpinolene	13.86 (11.31–16.58)	15.32 (12.67–18.19)	10.62 (8.24–13.58)
Terpinyl acetate	72.39 (65.05–80.55)	96.32 (89.89–103.04)	58.27 (52.60–64.82)
Thymol	11.45 (10.12–12.80)	11.72 (9.77–13.87)	10.79 (9.50–12.31)
Temephos *^b^*	0.010 (0.009–0.011)	0.015 (0.013–0.018)	0.013 (0.010–0.016)
Fenthion *^b^*	0.012 (0.010–0.015)	0.022 (0.019–0.024)	0.027 (0.023–0.031)

*^a^* Confidence limit. *^b^* Commercially available synthetic larvicides.

The toxic effects of PD-HD constituents and its structurally related compounds on the three mosquito species were similarly compared. Against the larvae of *Ae. albopictus*, the most toxic compound was *δ*-3-carene (LC_50_, 9.33 mg/L), followed by (+)-limonene, thymol, *α*-terpinolene, (1*S*)-(−)-*β*-pinene, *γ*-terpinene, *α*-phellandrene, *α*-terpinene and (1*R*)-(+)-*β*-pinene, ranging in their LC_50_ values from 10.77 to 23.44 mg/L (Table 2).

Thymol was the most toxic compound against larval *Ae. aegypti* (LC_50_, 11.72 mg/L), followed by *δ*-3-carene, (+)-limonene, *α*-terpinolene, (1*S*)-(−)-*β*-pinene, *α*-phellandrene, *γ*-terpinene, geranyl acetate, (1*R*)-(+)-*β*-pinene and *α*-terpinene, ranging in their LC_50_ values from 12.10 to 24.85 mg/L (Table 2).

As for the larvae of *Cx. p. pallens*, *δ*-3-carene showed the highest toxicity (LC_50_, 10.11 mg/L), followed by *α*-terpinolene, (+)-limonene, thymol, *α*-phellandrene, (1*S*)-(−)-*β*-pinene, *γ*-terpinene, *α*-terpinene and (1*R*)-(+)-*β*-pinene, ranging in their LC_50_ values from 10.62 to 24.02 mg/L (Table 2).

Among the compounds not specified here, some exhibited moderate toxicity (100 mg/L > LC_50_ > 25 mg/L) and some showed low toxicity (150 mg/L > LC_50_ > 100 mg/L), while the others were considered to have toxicity of insignificant magnitudes (LC_50_ > 150 mg/L).

Chi-square values for each of the determined LC_50_ values are given in a separate table (Table 3).

**Table 3:** Chi-square values for the determined LC_50_ values.

Compound	χ^2^ for LC50_ (*Ae. albopictus*)_	χ^2^ for LC_50 (*Ae. aegypti*)_	χ^2^ for LC_50 (*Cx p. pallens*)_
PD-HD	3.45	2.19	2.32
*α*-humulene	1.73	2.41	2.82
*α*-terpinene	1.82	2.09	2.52
Carveol	1.06	3.21	4.29
Nerol	1.59	1.20	2.95
(*S*)-(+)-carvone	5.07	2.91	3.27
(+)-*β*-citronellol	3.89	3.46	1.92
(−)-*β*-citronellol	3.47	3.82	4.62
(+)-citronellal	4.10	2.48	3.70
(−)-citronellal	2.40	1.37	4.19
(−)-myrtenal	2.22	2.43	4.09
(−)-cis-myrtanol	3.42	5.66	3.86
(−)-myrtenol	4.09	2.22	3.87
Carvacrol	5.05	1.01	1.93
(−)-verbenone	2.98	1.08	2.96
(*S*)-cis-verbenol	2.61	5.03	2.04
*o*-cymene	3.00	3.41	2.10
*m*-cymene	2.70	2.97	3.59
Aromadendrene	2.29	2.10	1.47
Borneol	3.41	3.99	3.70
Bornyl acetate	2.87	2.47	3.11
Camphene	4.01	1.08	3.42
Camphor	2.24	1.54	1.71
*δ*-3-carene	2.24	1.03	1.81
*β*-caryophyllene	2.85	1.09	5.42
Caryophyllene oxide	5.11	4.13	3.04
1,8-cineole	3.57	3.37	2.88
Citral	1.64	1.94	3.91
*p*-cymene	2.32	3.34	2.66
Geraniol	4.13	3.47	1.38
Geranyl acetate	2.97	2.13	2.47
(+)-limonene	1.98	2.69	2.67
Linalool	1.04	4.31	4.46
Linanyl acetate	2.19	3.20	4.96
Menthol	2.64	2.18	2.67
Myrcene	3.95	1.59	5.38
*α*-phellandrene	4.19	3.51	4.41
(1*R*)-(+)-*α*-pinene	1.87	1.28	1.83
(1*S*)-(−)-*α*-pinene	2.60	2.25	2.93
(1*R*)-(+)-*β*-pinene	1.87	1.47	1.76
(1*S*)-(−)-*β*-pinene	5.28	3.71	1.61
*γ*-terpinene	1.65	2.84	3.51
*α*-terpineol	3.79	2.36	4.15
(+)-terpinen-4-ol	2.89	3.77	2.56
(−)-terpinen-4-ol	1.24	2.36	1.75
*α*-terpinolene	1.11	1.35	1.62
Terpinyl acetate	1.28	3.12	2.75
Thymol	1.12	2.27	2.31
Temephos *^a^*	1.64	2.97	2.24
Fenthion *^a^*	3.62	4.46	3.03

*^a^* Commercially available synthetic larvicides.

### 3.2. Field Trials

**Figure 2:**
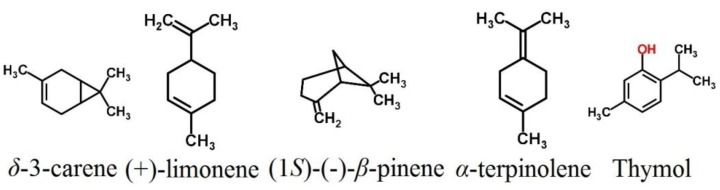
Chemical structures of the five most active test compounds that were used in semi-fieldbioassays.

The toxicity of PD-HD and 5 of its constituents/structurally related compounds against the field-collected larval *Cx. p. pallens* was evaluated and compared with the results observed in previous bioassays conducted with laboratory-reared *Cx. p. pallens* (Table 4). The performance of temephos and fenthion in semi-field bioassays was also recorded. Despite high levels of resistance that the field-collected larvae have developed toward temephos and fenthion (resistance ratios of 129.23 and 115.56 respectively) as compared to the laboratory-reared ones, PD-HD and its constituents exhibited potency of similar magnitudes against both field-collected and laboratory-reared larvae. Detailed chemical structures of the five most toxic constituents/structurally related compounds used in semi-field bioassays are shown in Figure 2.

**Table 4:** Toxicity of *Pinus densiflora* hydrodistillate (PD-HD), five selected compounds and two commercially available synthetic larvicides against early third-instar larvae of field-collected and laboratory-reared *Cx. p. pallens.*

	Field-collected	Laboratory-reared	
Compound	LC_50_, mg/L	LC_50_, mg/L	Resistance ratio
PD-HD	21.02	22.36	0.94
*δ*-3-carene	12.67	10.11	1.25
(+)-limonene	10.54	10.76	0.98
(1*S*)-(−)-*β*-pinene	16.80	16.17	1.04
*α*-terpinolene	13.92	10.62	1.31
Thymol	11.25	10.79	1.04
Temephos *^a^*	1.68	0.013	129.23
Fenthion *^a^*	3.12	0.027	115.56

*^a^* Commercially available synthetic larvicides.

### 3.3. AChE Inhibition

The AChE inhibitory activity of the selected compounds for each of the three mosquito species is recorded in Table 5. In general, thymol and *δ*-3-carene were found out to be the strongest inhibitors of AChE in all three species (IC_50_ < 2 mM). The other compounds exhibited either moderate (IC_50_ < 15 mM) or low (IC_50_ > 15 mM) AChE inhibitory activity.

**Table 5:** *In vitro* inhibition of acetylcholinesterase (AChE) extracted from the heads of larval *Ae. albopictus*, *Ae. aegypti and Cx. p. pallens* by 10 selected compounds.

Compound	IC_50_, mM (*Ae. albopictus*)	IC_50_, mM (*Ae. aegypti*)	IC_50_, mM (*Cx. p. pallens*)
Thymol	1.48	1.96	1.57
*δ*-3-carene	1.20	1.45	1.90
*β*-caryophyllene	32.76	36.03	29.97
Citral	8.07	14.27	12.48
Menthol	20.67	18.62	25.78
1,8-cineole	62.45	74.33	68.26
Linalyl acetate	24.38	29.82	37.92
Carveol	43.02	49.14	42.10
(−)-myrtenal	23.73	34.03	31.62
*α*-humulene	47.59	58.11	62.32

## 4. Discussion

It is indeed widely acknowledged that certain plant extracts and their constituents have the potential of being developed into products that are suitable for integrated arthropod disease vector management because they can be selective and environmentally non-persistent, unlike the synthetic pesticides currently in use, and have very few toxic effects on non-target organisms [8,9]. They can also be used in conjunction with various other means of biological control [8]. Essential oils extracted from plants are made up of highly complex mixtures of oxygenated compounds (alcohols, aldehydes, ketones, phenols, oxides, esters and *etc.*) and hydrocarbons (mostly terpenoids) [21]. Their efficacy against various arthropod disease vectors is manifested in both behavioral (repellence and feeding deterrence) and physiological (acute toxicity and growth inhibition) aspects [8,9]. Certain plant extracts and their constituents have been well noted for their insecticidal activity against mosquitoes and proposed as possible alternatives to the synthetic mosquito insecticides currently in use. For example, plant extracts from eight different *Cinnamomum* species were reported to have significant larvicidal activity against dengue vector mosquitoes [22], and volatile compounds identified in the roots of *Asarum heterotropoides*, such as (−)-asarinin, methyleugenol and pellitorine, were shown to be effective against three species of mosquitoes [23].

The present study provides the first report of insecticidal activity of *Pinus densiflora* hydrodistillate and its active compounds against mosquitoes. In the present study, potent toxicity against *Ae. albopictus*, *Ae. aegypti* and *Cx. p. pallens* was obtained from both PD-HD and many of its constituents and structurally related compounds, including *δ*-3-carene, (+)-limonene, thymol, *α*-terpinolene, (1*S*)-(−)-*β*-pinene, *γ*-terpinene, *α*-phellandrene, *α*-terpinene and (1*R*)-(+)-*β*-pinene. No significant difference in toxicity against the three mosquito species was observed. Although PD-HD and its constituents were of much lower toxicity than the synthetic larvicides, temephos and fenthion, they were found to be equally effective against both laboratory-reared and field-collected larvae, the latter of which have developed high levels of resistance to currently used synthetic larvicides. Thus, it is safe to conclude that PD-HD holds much promise for the development of alternative larvicides that can be effective even against the insecticide-resistant mosquito populations.

It should be noted that some of the compounds tested in this study exhibited potency of different magnitudes from those observed in other studies. In a previous study conducted by Eleni *et al.* [24], for instance, linalyl acetate was found to possess higher potency than in the present study and according to a study by Giatropoulos *et al.* [25], (+)-limonene exhibited lower potency. Such differences in LC_50_ values may be accounted for by considering the fact mosquitoes used in the experiments, although they might belong to the same species, were of different strains.

Moreover, in light of the above-mentioned concerns that several mosquito species are showing signs of increasing resistance to currently used insecticides, investigations on the modes of action of natural insecticidal products are of critical importance to the development of a successful resistance management strategy. As such, several active compounds identified in PD-HD were tested for their AChE inhibitory activity (Table 5) in the hope that results from such experiments would shed new light on the modes of action of the compounds. The observed AChE inhibitory activity (IC_50_) was then correlated with the larvicidal activity (LC_50_). Results are summarized in Table 6. The correlation coefficients were 0.617, 0.604 and 0.720 for *Ae. albopictus*, *Ae. aegypti* and *Cx. p. pallens* respectively. Based on these results, there seems to be a relatively strong positive correlation between LC_50_ and IC_50_ of the test compounds. Although some studies have failed to find a direct correlation between insect toxicity and AChE inhibition by terpenoids [26], the findings of the present study are consistent with and thus corroborate the report of Ryan and Byrne [27] that there is a significant relationship between insecticidal and AChE (extracted from electric eels) inhibitory activities of terpenoids. Although there is not yet enough evidence to come to a definite conclusion, it is likely that AChE is the major site of action for the compounds tested. In the future, more detailed experiments are indeed needed to fully understand the exact modes of action of these compounds.

**Table 6:** Correlation analysis between LC_50_ and IC_50_ values of 10 selected compounds.

Species	Correlation coefficient, *r*
*Ae. albopictus*	0.617
*Ae. aegypti*	0.604
*Cx. p. pallens*	0.720

## 5. Conclusions

In conclusion, PD-HD and compounds described herein could be of practical use as larvicides in the control of disease vector mosquito populations. In order for these test materials to be developed as novel larvicides, further research is required to validate their safety to mammalian and human health, non-target aquatic organisms and the surrounding environment. In addition, formulations need to be investigated that can improve potency and stability, thereby reducing the costs of production involved.
